# Extensively drug-resistant Salmonella typhi Infection in Adults; Experience from A Tertiary Care Hospital

**DOI:** 10.12669/pjms.40.6.8384

**Published:** 2024-07

**Authors:** Afsheen Mahmood, Fawad Rahim, Said Amin, Mohammad Noor

**Affiliations:** 1 Afsheen Mahmood, FCPS. Department of Medicine, Medical Teaching Institute, Hayatabad Medical Complex, Peshawar, Pakistan; 2 Fawad Rahim, FCPS. Department of Medicine, Medical Teaching Institute, Hayatabad Medical Complex, Peshawar, Pakistan; 3 Said Amin, FCPS. Department of Medicine, Medical Teaching Institute, Hayatabad Medical Complex, Peshawar, Pakistan; 4 Mohammad Noor, FRCPE. Department of Medicine, Medical Teaching Institute, Hayatabad Medical Complex, Peshawar, Pakistan

**Keywords:** Extensively drug-resistant Salmonella typhi, XDR, Typhoid fever, Salmonella typhi, Enteric fever

## Abstract

**Objectives:**

This study aimed to determine the epidemiology, clinical features, and complications of extensively drug-resistant Salmonella typhi (XDR S. typhi) infection in adults.

**Method:**

This cross-sectional study enrolled adults with culture-proven XDR S. typhi admitted to Hayatabad Medical Complex, Peshawar from 1^st^ March to 10^th^ September 2022. Their demographic characteristics, clinical features, treatment, and complications were recorded.

**Results:**

Out of 84 patients, 68 (80.9%) were male. The mean age of enrolled patients was 25.2 ± 11.3 years. The mean duration of fever at the time of admission was 13.6 ± 8.2 days, respectively. The most common symptom was loose stools (n=25, 29.8%). Most of the patients (n=69, 82.1%) had received empirical treatment before hospitalization. The majority of the patients (n=42, 50%) received meropenem and a combination of meropenem and azithromycin (n=35, 41.7%) during the study. The time to defervescence for both regimens was similar. Five patients (6%) developed complications of enteric fever. There was no mortality among the participants.

**Conclusions:**

Diarrhea was the most common associated clinical feature in XDR typhoid fever. Most of the patients received meropenem alone or in combination with azithromycin with a comparable time to defervescence. The majority of the patients recovered uneventfully and there was no mortality among the study participants.

## INTRODUCTION

Waterborne infections such as typhoid fever, viral hepatitis, and amoebic & bacillary dysentery are common in developing countries like Pakistan, where clean drinking water and proper disposal of sewage are lacking. Typhoid fever affects 21.6 million people globally and is responsible for 250,000 deaths yearly.^1^ It is on the rise in Pakistan amidst the COVID-19 pandemic, with 20,000 cases reported during the first 10 days of June 2020.^2^

Typhoid fever is a major public health issue in poor and developing countries like Pakistan. Although antibiotics significantly mitigate the mortality and morbidity of typhoid, with the isolation of extensively drug-resistant (XDR) Salmonella typhi (S. typhi) in Hyderabad, Sindh, Pakistan, there is a fear that we will end up in the pre-antibiotic era.^3^ Federal Disease Surveillance and Response Unit, Field Epidemiology & Disease Surveillance Division, National Institute of Health (NIH) reported a total of 22 571 cases of typhoid fever from 1^st^ November 2016 to 16^th^ February 2020 in the province of Sindh, Pakistan. Out of these, there were 16000 cases of XDR S. typhi, and the majority were from Karachi.^4^ The outbreak is not only a local concern, but travelers from Pakistan to the United States of America (USA), the United Kingdom, and Canada have been found to be infected with XDR S. typhi. Extensively drug-resistant S. typhi has been found among people who did not travel outside the USA.^5^

Salmonella typhi, including the XDR strain, causes an acute febrile illness similar to malaria, dengue fever, chikungunya, and viral hepatitis, which are not uncommon in this region. Other clinical features include abdominal pain and loose motions, relative bradycardia, abdominal tenderness, hepatomegaly, splenomegaly and rose spots, particularly in fair-skinned people. Acute hepatitis, acute appendicitis, and abscesses and infarcts in spleen and kidneys due to XDR S. typhi have been reported.^6^,^7^ Extensively drug-resistant S. typhi is sensitive only to two classes of drugs, i.e., carbapenems and azithromycin. The outbreak is becoming a global threat with the isolation of an XDR S. typhi strain resistant to azithromycin in Bangladesh.^8^causative agents of typhoid and paratyphoid, have led to fears of untreatable infections. Of specific concern is the emerging resistance against azithromycin, the only remaining oral drug to treat extensively drug resistant (XDR Azithromycin and carbapenems, especially meropenem, have been indiscriminately used in Pakistan during the COVID-19 pandemic. This irrational use may lead to the emergence of a strain of S. typhi resistant to these antibiotics.^9^

Pakistan is a densely populous country with diverse ethnicity and culture across its five provinces. Extensively drug-resistant S. typhi was first reported in Pakistan from the southern province of Sindh in 2016. Subsequently, outbreaks were reported from the capital territory of Islamabad (2019) and the central province of Punjab (2020).^3^with Pakistan's inhabitants at most risk amongst Asian countries where typhoid remains prevalent. Decades of indiscriminate antibiotic usage has driven the evolution of multidrug-resistant strains and more recently, extensively drug-resistant (XDR In addition, there is an ongoing outbreak of XDR S. typhi in Khyber Pakhtunkhwa province. Given its high cost of treatment in our resource constraint setting and the associated complications, the emergence and spread of XDR S. typhi is a significant public health challenge.

Most of the studies on XDR S. typhi in Pakistan have focused on the pediatric population.^9^-^11^ There is a notable knowledge gap of its clinical course and consequences in adult patients. We aimed to fill this gap by determining the epidemiological factors, clinical features, and complications of XDR S. typhi in the adult population admitted in a tertiary care hospital in Peshawar, Pakistan. The findings of the study will help clinicians make informed decisions in the management of patients infected with XDR S. typhi.

## METHOD

This cross-sectional study was conducted in the Department of Medicine, Hayatabad Medical Complex, Peshawar, Pakistan between 1^st^ March 2022 to 10^th^ September 2022. Among patients aged 14 years and above admitted for acute febrile illness, those with blood culture-positive XDR S. typhi were included in the study using consecutive sampling technique after informed written consent. S. typhi was isolated from blood cultures on MacConkey agar, and antibiotic sensitivity testing was carried out using disk diffusion method. S. typhi were labelled as XDR when they were found resistant to fluroquinolones and third generation cephalosporins.

### Ethical Approval:

The institutional/ethical review board of Khyber Girls Medical College, Peshawar, Pakistan, approved the study (Letter No. 726/Estt, dated 16.02.2022).

All cases were notified to the provincial infectious disease surveillance department, Khyber Pakhtunkhwa, Pakistan. Additionally, all the patients were screened for malarial parasite, dengue fever, hepatitis A, B, C, D and E viruses, and Leptospira. All patients received antibiotics as per culture report, with the choice of antibiotics used (meropenem (20-40mg/kg every eight hours), azithromycin (20mg/kg/day), or a combination of the two) left at the discretion of the attending physician. Some patients were also offered dexamethasone at the attending physician’s discretion. Every patient was assessed daily for temperature, pulse, blood pressure, worsening abdominal pain, melena, abdominal tenderness, jaundice, and other complications of typhoid fever. The outcome was grouped into two: patients who developed complications related to typhoid fever and those who did not. Defervescence was defined as an absence of fever for 72 hours without the use of an antipyretic.

The demographic parameters, clinical features, empirical treatment before admission, the antibiotic regimens used for the treatment, the use of dexamethasone, the time to defervescence, and the complications developed during the course of the illness were recorded. The study is reported per The Strengthening the Reporting of Observational Studies in Epidemiology (STROBE) Statement: guidelines for reporting observational studies.

The data were analyzed using Statistical Package for the Social Sciences, version 21. Mean and standard deviation were calculated for age, the duration of fever at the time of admission, and the time to defervescence. Frequency and percentage were calculated for age groups, gender, associated clinical features, empirical treatment before admission, antibiotic regimens, use of dexamethasone, and complications. Continuous variables were assessed for normality using the Shapiro-Wilks test. The significance of differences in continuous and categorical variables between groups was assessed using the Mann-Whitney U test and one-way ANOVA, and the Chi-square test, respectively. A p-Value ≤ 0.05 was considered significant.

## RESULTS

Out of 84 patients, majority (n=68, 80.9%) were male. The mean age and duration of fever at the time of admission were 25.2 ± 11.3 years and 13.6 ± 8.2 days, respectively. Most of the patients were in the age group 16 - 25 years (51.2%). In addition to fever, the most common symptom was loose stools (n=25, 29.8%). Sixty-nine (82.1%) patients had received empirical treatment before admission. The majority of the patients (n=42, 50%) received only meropenem. Dexamethasone was used in 28.6% of cases. Only five patients (6%) developed complications of enteric fever. Demographic and clinical parameters of the study participants are summarized in (Table-I).

**Table-I (a):** Demographic and clinical characteristics of the study population (n=84).

Parameters	No. (%)
***Age groups***	
Up to 15 years	13 (15.5%)
16 - 25 years	43 (51.2%)
26 - 35 years	14 (16.7%)
36 - 45 years	8 (9.5%)
Older than 45 years	6 (7.1%)
***Gender***	
Male	68 (80.9%)
Female	16 (19.1%)
***Associated symptoms***	
Headache	08 (9.5%)
Body Aches	20 (23.8%)
Abdominal Pain	20 (23.8%)
Loose stools	25 (29.8%)
Vomiting	20 (23.8%)
***Empirical treatment before admission***	
Yes	69 (82.1%)
No	15 (17.9%)
***Antibiotics regimen***	
Meropenem alone	42 (50%)
Azithromycin alone	07 (8.3%)
Meropenem & azithromycin	35 (41.7%)
***Dexamethasone***	
Yes	24 (28.6%)
No	60 (71.4%)
***Complications***	
No	79 (94%)
Gastrointestinal hemorrhage	01 (1.2%)
Hepatitis	01 (1.2%)
Intestinal perforation	01 (1.2%)
Septic shock	00
Encephalopathy	01 (1.2%)
Visceral abscess	01 (1.2%)

SD: Standard deviation.

**Table-I (b):** Demographic and clinical characteristics of the study population (n=84).

Parameters	Mean ± SD
Age	25.2 ± 11.3
Duration of fever at the time of admission	13.6 ± 8.2
***Defervescence time (Days)***	
Meropenem alone	7.0±1.4
Azithromycin alone	6.7±1.4
Meropenem & azithromycin	6.9±1.3

Compared to patients without abdominal pain at admission, those with pain were significantly younger (26.7 ± 11.9 versus 20.3 ± 7.4, p=0.013). However, there were no significant differences in the mean age with respect to other symptoms associated with fever. Similarly, the mean duration of fever did not differ significantly among patients with or without the other related symptoms. A significantly higher proportion of males presented with loose stools (35.3% vs 6.2%, p=0.022). There was no gender-wise difference in the frequency of other associated symptoms (Table-II).

**Table-II:** Relationship between age, duration of fever and gender, and the associated symptoms.

		Age		Duration of fever		Gender		

		Mean ± SD (years)	p-Value	Mean ± SD (days)	p-Value	Male No. (%)	Female No. (%)	p-Value
Headache	No	25.0 ± 11.6	0.344	13.5 ± 8.2	0.818	62 (91.2%)	14 (87.5%)	0.652
	Yes	26.5 ± 8.6		14.4 ± 8.5		06 (8.8%)	02 (12.5%)	
Body Aches	No	23.9 ± 10.5	0.065	12.7 ± 7.4	0.186	52 (76.5%)	12 (75%)	0.901
	Yes	29.1 ± 13.0		16.6 ± 10.1		16 (23.5%)	04 (25%)	
Abdominal Pain	No	26.7 ± 11.9	0.013	14.0 ± 8.5	0.568	54 (79.4%)	10 (62.5%)	0.153
	Yes	20.3 ± 7.4		12.4 ± 7.2		14 (20.6%)	06 (37.5%)	
Loose Stools	No	25.2 ± 12.0	0.721	14.1 ± 8.5	0.457	44 (64.7%)	15 (93.8%)	0.022
	Yes	25.0 ± 9.5		12.5 ± 7.6		24 (35.3%)	01 (6.2%)	
Vomiting	No	25.8 ± 11.7	0.286	14.0 ± 8.2	0.283	52 (76.5%)	12 (75%)	0.901
	Yes	23.0 ± 9.4		12.5 ± 8.4		16 (23.5%)	04 (25%)	

SD: Standard deviation.

There were no differences in the mean age and duration of fever among patients who received azithromycin, meropenem, or a combination of the two antibiotics. Likewise, compared to patients who received dexamethasone, the mean age, and duration of fever of those who did not receive dexamethasone were not significantly different. Azithromycin was advised for a significantly higher proportion of females (25% versus 4.4%, p=0.007). In contrast, no gender-wise differences were observed in the use of meropenem, the combination of meropenem and azithromycin, and dexamethasone (Table-III).

**Table-III:** Relationship between age, duration of fever and gender, and the medications used.

		Age		Duration of fever		Gender		

		Mean ± SD (years)	p-Value	Mean ± SD (days)	p-Value	Male No. (%)	Female No. (%)	p-Value
Azithromycin alone	No	25.2 ± 11.7	0.517	13.5 ± 8.0	0.941	65 (95.6%)	12 (75%)	0.007
	Yes	24.6 ± 4.8		15.3 ± 11.2		03 (4.4%)	04 (25%)	
Meropenem alone	No	25.8 ± 9.9	0.188	14.3 ± 7.8	0.264	34 (50%)	08 (50%)	1.000
	Yes	24.6 ± 12.6		13.0 ± 8.6		34 (50%)	08 (50%)	
Meropenem & Azithromycin	No	24.6 ± 11.7	0.331	13.3 ± 8.9	0.240	37 (54.4%)	12 (75%)	0.133
	Yes	26.0 ± 10.6		14.1 ± 7.2		31 (45.6%)	04 (25%)	
Dexamethasone	No	23.5 ± 9.2	0.184	14.9 ± 9.1	0.168	48 (70.6%)	12 (75%)	0.725
	Yes	29.3 ± 14.7		10.5 ± 4.2		20 (29.4%)	04 (25%)	

SD: Standard deviation.

## DISCUSSION

The history of S. typhi infections and emerging treatment options spans over decades. Chloramphenicol was identified as an antimicrobial for typhoid fever in the late 1940s. Two years later, however, clinical reports indicated Salmonella have become resistant to chloramphenicol. Resistance to fluoroquinolones developed in 2015.^12^ Pakistan has the second highest burden of typhoid fever in the world and has been in the spotlight regarding drug resistance.^13^ The first case of XDR S. typhi was isolated in Pakistan in 2016. Most of the published research regarding XDR S. typhi has focused on the pediatric population.^9^,^10^,^14^ Emerging resistance to S. typhi in Pakistan is one of the key issues and the main dangers faced by the pediatric population. It is sparing neither any region nor any age group.^14^

The mean age of the study participants was 25.2±11.3 years while Fida et al. from Lahore, Pakistan reported a mean age of 31±9 years.^15^ The difference could be that the sample reported by Fida et al. was limited to the military recruits and their families and, unlike the sample of this study, did not represent the general population.^15^ Moreover, the mean age in other studies from the region ranged from 8 to 14 years.^9^,^10^

Most of the patients were male (n=68, 80%). A similar finding had been reported by Fida et al.^15^ Male predominance had been a consistent finding across all age groups in studies reported from this region.^9^,^10^,^14^ The male predominance could be due to the cultural norms where the males are more likely to go out for work and dine out. Thus, they get infection from unhygienic food and water sources. In addition, for similar reasons, females are less likely to report to healthcare facilities. In contrast, most of the patients in an outbreak reported from China were female.^16^ The reason for the high proportion of females in the Chinese study was that the outbreak was reported from an apartment and not from the general population.

Loose stools were observed in 25% (n=29),abdominal pain in 20%(n=23), vomiting in 23%(n=20) and headache in 9.5% (n=8) patients in this study. Fida et al reported similar findings.^15^ Shahid et al, reported comparable results for loose stools (26%) and vomiting (36%) in the pediatric age group.^10^ Huges MJ et al, from the United States of America found abdominal pain in 30% of travelers with XDR S. typhi.^5^ In contrast, Wang Y et al, from China reported diarrhea in 47.8% of patients with XDR S. typhi.^16^

The results of current study showed that 82.1% (n = 69) of patients were treated empirically before admission, while Sonia et al, from Hyderabad reported 64% (n = 52).^11^ Self and over-the-counter medications have been a common practice in Pakistan and vary with location, culture, and literacy level.^17^ This difference could be due to the low literacy rate in Khyber Pakhtunkhwa from where the study is reported (55.1% vs 61.8%).^18^

Meropenem and azithromycin are the last weapons in the armory of antibiotics against XDR S. typhi. In this cross-sectional study, 50% (n=42) patients received meropenem alone, 41.7% (n=35) meropenem along with azithromycin, and 8.5% (n=7) azithromycin alone. In the absence of uniform guidelines for selecting antibiotics for XDR S. typhi, there has been a discrepancy in the choice of antibiotic as the selection was left to the discretion of the attending physician.^9^-^11^ While Fida et al. had not shared the choice of antibiotics used for XDR S. typhi.^15^

In this study, the defervescence time for either azithromycin or meropenem or a combination of meropenem and azithromycin was comparable. The study by Qureshi et al also revealed similar defervescence times of all three regimens.^11^ This signifies the similar efficacy of all three regimens throughout Pakistan.

In the adult population, we experienced favorable defervescence by adding dexamethasone (n=42, 28.6%) to the standard treatment. However, there are conflicting reports on the use of steroids in severe typhoid fever.^19^ This warrants further clinical trials in adults with XDR S. typhi infection.

The frequency of hepatitis was similar in this study compared to the study reported by Fatima et al.^20^ In contrast, Herekar et al. from Karachi reported a high proportion of patients with hepatitis (14%).^21^ Shahid et al. and Fatima et al. from Karachi observed a high rate of intestinal perforation in the pediatric population, 13% and 23%, respectively.^10^,^20^ In contrast to this study, Fida et al. have reported 11.5% cases of ileal perforation leading to peritonitis despite a similar duration of fever at the time of admission (13.6±8.2 versus 13.5±6.6 days) and similar antibiotic regimens.^15^ Dexamethasone was used in 28.6% of patients in this study. Whether steroids prevent ileal perforation warrants further controlled trials in adults with XDR S. typhi infection.

There was no mortality in the study population, and similar finding had been reported by Fida et al.^15^ In contrast, Qureshi et al, Fatima et al, and Herekar et al, reported mortality of 1.55%, 1.8%, and 1.9

### Strength & Limitations of the Study:

The main strength of this study was the largest sample size of adult patients with XDR S. typhi infection. Additionally, the sample was drawn from the community. We consider the lack of long-term follow-up after discharge from the hospital as a limitation of the study. Moreover, this study was limited to in-patient cases of XDR S. typhi only, therefore, the results cannot be generalized to the population at large.

## CONCLUSION

The majority of the cases of XDR S. typhi were adolescent males who presented approximately two weeks after the onset of fever. Apart from fever, diarrhea was the most common associated clinical feature. Most of the patients received meropenem alone or in combination with azithromycin. The majority of the patients recovered uneventfully and there was no mortality among the study participants. Extensively drug-resistant S. typhi should be included in the differential diagnosis of acute febrile patients, particularly with gastrointestinal manifestations in endemic regions like Pakistan. It should also be considered among travelers from the endemic regions. Appropriate antibiotics for the cases and vaccination for the public at large are the need of the hour.
